# The effects of Hartcoach, a life style intervention provided by telephone on the reduction of coronary risk factors: a randomised trial

**DOI:** 10.1186/1471-2261-12-47

**Published:** 2012-06-26

**Authors:** Chantal J Leemrijse, Liset van Dijk, Harald T Jørstad, Ron J G Peters, Cindy Veenhof

**Affiliations:** 1 Netherlands institute for health services research, Utrecht, The Netherlands; 2 Department of Cardiology, Academic Medical Centre, Amsterdam, The Netherlands

**Keywords:** Cardiovascular disease, Secondary prevention, Self management, Nurse led telephonic intervention

## Abstract

**Background:**

Cardiovascular disease (CVD) is the leading cause of death worldwide. Secondary prevention is essential, but participation rates for cardiac rehabilitation are low. Furthermore, current programmes do not accomplish that patients with CVD change their lifestyle in a way that their individual risk factors for recurrent events decrease, therefore more effective interventions are needed. In this study, the effectiveness of the Hartcoach-programme, a telephonic secondary prevention program focussing on self management, is studied.

**Methods/design:**

A multicenter, randomised parallel-group study is being conducted. Participants are 400 patients with acute myocardial infarction (STEMI, NSTEMI,) and patients with chronic or unstable angina pectoris (IAP). Patients are recruited through the participating hospitals and randomly assigned to the experimental group (Hartcoach-programme plus usual care) or the control group (usual care).

The Hartcoach-programme consists of a period of six months during which the coach contacts the patient every four to six weeks by telephone. Coaches train patients to take responsibility for the achievement and maintenance of the defined target levels for their particular individual modifiable risk factors. Target levels and treatment goals are agreed by the nurse and patient together. Data collection is blinded and occurs at baseline and after 26 weeks (post-intervention). Primary outcome is change in cardiovascular risk factors (cholesterol, body mass index, waist circumference, blood pressure, physical activity and diet). Secondary outcomes include chances in glucose, HbA1c, medication adherence, self-management and quality of life.

**Discussion:**

This study evaluates the effects of the Hartcoach-programme on the reduction of individual risk factors of patients with CVDs. Patients who are not invited to follow a hospital based rehabilitation programme or patients who are unable to adhere to such a programme, may be reached by this home based Hartcoach-programme. If positive results are found, the implementation of the Hartcoach-programme will be extended, having implications for the management of many people with CVD.

**Trial registration:**

NTR2388

## Background

Although many cardiovascular diseases (CVDs) can be treated or prevented, an estimated 17.3 million people die of CVDs each year, representing 30% of all global deaths [1]. Survivors of a heart attack or stroke are at high risk of recurrences and at high risk of dying from them as well. Therefore, secondary prevention is essential. The purpose of the present study is to evaluate the effects of a home-based secondary prevention programme focussing on self management, provided by telephone.

The main controllable risk factors for cardiovascular disease include elevated cholesterol, high blood pressure, smoking, overweight, diabetes mellitus and a sedentary lifestyle [2]. Research has shown that changing these risk factors to appropriate targets significantly reduces the risk of recurrent coronary heart diseases, decreases the need of interventional procedures, and improves quality of life [3]. However, a treatment gap between scientific evidence and daily practice exists and current programmes do not accomplish that patients with coronary artery disease change their lifestyle in a way that their individual risk factors for recurrent coronary heart disease decrease [4-6]. Therefore, more effective lifestyle management interventions and more proactive management of the disease are needed [7].

With a chronic disease such as CVD, the patient should become a partner in the health care process [8,9]. Complementary to the doctor’s general knowledge, the patient can provide individual information on his health status, risk factors, and changes in illness patterns. Since both sources of information are important for taking adequate treatment decisions, the patient should be actively involved in his treatment and contribute to almost every decision. To enhance the ability of patients to actively participate in their own healthcare and to accomplish a central role, guidance in self management is needed [10]. Adequate self management requires knowledge of one’s own individual risk factors, strategies to influence those factors and the ability to cope with emotions such as fear and frustration [8,9].

Patients with an acute coronary event or unstable angina pectoris are usually admitted to the hospital. In the Netherlands, the average hospital lengths of stay for people with coronary diseases diminished between 1995 and 2007 from almost nine days to less than 6 days [11]. One of the impacts of this reduced hospital stay may be the reduction in time for nurses to offer emotional support and to provide the patient and his family with pre discharge education on risk factors and the relation with lifestyle. People with cardiac disease report dissatisfaction with their education upon discharge and a lack of professional support [12,13]. Although some patients participate in a hospital based rehabilitation programme including life style intervention and self management after discharge, the availability, content and extensiveness of these programmes differ strongly per hospital. Furthermore, not all patients may be able to visit the hospital regularly. Therefore, alternative models are needed to increase access to secondary prevention and guidance for self management.

In a meta-analysis on different types of secondary prevention programmes for coronary heart disease including self management strategies, Clark et al. (2010) showed that home-based interventions compared with usual care significantly improved quality of life, systolic blood pressure, smoking cessation, total cholesterol and depression [14]. Neubeck et al. (2009) concluded from a systematic review on telehealth interventions including telephone, internet and videoconference communication, that telephone based interventions were the most effective in reducing the patient’s total cholesterol, systolic blood pressure and low and high lipoprotein [15]. Possibly, a home-based intervention provided by telephone could help to increase access to secondary prevention programmes and narrow the gap between evidence and practice.

A programme that provides home-based education and professional support for self management after discharge from the hospital is the COACH-programme (Coaching Patients On Achieving Cardiovascular Health (COACH). COACH was developed in Australia [16-18] and is provided by specifically trained nurses via the telephone. In this intervention, patients are trained to take responsibility for the achievement and maintenance of the defined target levels for their particular individual modifiable risk factors. In addition, medication adherence is stimulated. Target levels and treatment goals are agreed upon by the nurse and patient. Such collaborative goal setting and defining the targets for treatment is an effective method to promote self management [19]. The regular telephone contacts not only offer the patient an accessible opportunity to ask health related questions, but may also provide the patient with emotional support.

In Australia, the effects of the COACH-programme have been evaluated. In a randomized clinical trial, the total cholesterol levels of the group of patients who were guided according to the COACH-programme were significantly lower compared to the cholesterol levels of the patients who received usual care alone. Furthermore, the COACH-programme resulted in a greater reduction in body weight, BMI, dietary intake of total fat, saturated fat, and anxiety level. Patients who were coached reported better health and mood after six months treatment, and reported more regular walking for exercise [17].

In the Netherlands, Health insurance company Achmea has introduced the COACH concept. Since there are differences in health care system and cultural background between both countries, there is a need to replicate the effectiveness study of the Australian programme in the Dutch situation. In addition, in the Netherlands, the coaches providing this intervention are all employed by Achmea while in Australia coaches were employed by the hospital. To avoid confusion with another Dutch programme specifically developed for patients with heart failure, the COACH-programme is renamed as “Hartcoach-programme”. The Hartcoach-programme is expected to have a beneficial effect on reducing risk factors and health of patients with CVD in the Netherlands.

## Methods

### Objectives of this research

Primary aim: To investigate the effect of the Hartcoach-programme in addition to usual care on the individual risk factors cholesterol, body mass index, waist circumference, blood pressure, physical activity and diet in patients with a coronary event (ACS or stable angina which PCI and / or CABG).

Secondary aim: To see to what extent the Hartcoach-programme affects glucose, HbA1c, smoking, medication adherence, self-management, anxiety, depression and quality of life, compared with usual care alone.

## Design

A multicenter, randomised parallel-group study will be conducted. The experimental group (Hartcoach-programme plus usual care) will be compared with a control group (usual care only). Four-hundred patients diagnosed with CVD in five hospitals will be included, 200 in each group. Patients are recruited through the participating hospitals and are (in each hospital) randomly assigned to one of the two groups via a computerized procedure at the coordinating research centre (NIVEL). The Hartcoach study had been approved by the Medical Ethics Board of the University of Amsterdam, The Netherlands.

### Participants

Participants are patients aged between 18 and 80 years with documented evidence of coronary heart disease who have been hospitalized less than 8 weeks before inclusion. This includes patients with an acute myocardial infarction (STEMI, NSTEMI,) and patients with chronic or unstable angina pectoris (IAP). Patients may have been treated with revascularization procedures, either coronary artery bypass graft surgery (CABG) or percutaneous coronary intervention (PCI) and/or with medication. Exclusion criteria are:

 planned surgery, percutaneous coronary intervention or other interventions;

 limited life-expectancy (< 2 years) according to the treating cardiologist;

 NYHA class III or IV heart failure;

 previous or current similar life style interventions (i.e. any intervention with multiple sessions focussing explicitly or exclusively on changing patients life style; cardiac rehabilitation in which occasionally advices according to life style are given is allowed);

 no telephone;

 insufficient control of the Dutch language.

#### *Recruitment*

All patients of the participating hospitals who meet the inclusion criteria are informed about the study by a specifically trained research assistant of the hospital. The patient informed consent information letter contains information about the nature of the study, the randomisation procedure and data collection at baseline and post test after 6 months. Treating specialists are instructed that the study will take place in their medical centre and are informed about the objective of the study. The patient’s general practitioner will be informed about the participation of their patient.

#### *Study setting*

The study concerns a home based secondary intervention programme provided per telephone. Patients are recruited from the Departments of Cardiology of several Medical centres in the Netherlands. All measurements (body length, weight, waist circumference, blood pressure, anamnesis and patients questionnaire) are performed by research nurses in these hospitals. Blood values are determined in the hospitals laboratory.

### Intervention

The Hartcoach-programme consists of a period of 6 months in which the coach contacts the patient every four to six weeks by telephone. The Dutch coaches are nurses who are working at the medical call centre of Achmea in Zwolle. They were trained in the spring of 2009 in Australia and additionally participated in a course on Motivational Interviewing in 2011.

Before starting the first coaching session, the baseline values for risk factors and medication use are established.

In each contact, a continuous quality improvement cycle is followed [17]:

1. Education – part 1. Asking questions to establish patients’ knowledge, attitude and beliefs about their risk factors. Questions are asked about medication, physical activity and dietary intake of saturated fat.

2. Education – part 2. The patient is informed and educated about those aspects for which his/her knowledge is insufficient.

3. Assertiveness training / empowerment. The patient is trained to be assertive in his/her relation with the doctor: to obtain the cholesterol level, to agree upon the target cholesterol level, to alter medication or doses if appropriate etc.

4. Goal setting. Negotiating of a plan of action and setting goals to be achieved by the next coaching session.

5. Reassessment at the next coaching session. Patient adherence to the negotiated plan of action is evaluated.

The coaches register per telephone call the date, topics that were discussed, advices given and agreements made. The patient receives a written letter with the most important advices and appointments summarized. Based on these reports, the researchers are able to verify whether the intervention is performed as intended. In daily practice, the coaching cycle is continued until target levels for risk factors are reached, usually in about 6 months. For the purpose of this study, the coaching cycle is set to 6 months exactly, in order to achieve similar post-treatment periods for all patients.

The experimental group will receive the Hartcoach-programme in addition to the usual care patients receive after they are discharged from the hospital. Usual care in the Netherlands differs per hospital and per patient, and can consist of visiting the cardiologist, general practitioner, physical therapist, dietician and/or taking medication. Usual care may also include cardiac rehabilitation. In case this rehabilitation mainly and specifically focuses on life style modification, the patient is excluded from the study. The control group receives usual care only. In a dairy, all patients register the amount and type of usual care they receive. In this way, insight is given in the usual care patients actually received and in case the amount and type of usual care differs between both groups, there will be controlled for it in the data analyses”.

After the post measurement after 6 months, patients from the control group are offered to participate in the Hartcoach-programme.

### Outcomes

All measurements for both the experimental- and the control group are performed at baseline after receiving the informed consent (T_0_) and before starting the Hartcoach-programme, and after 6 months (T_1_)^a^. Definitions of primary and secondary outcome measures are presented in Table 1. Targets presented in this table reflect the ideal outcomes. However, in the coaching sessions individualized and feasible targets are defined that may not always fully correspond with those ideal outcomes. For example, a patient may strive for less smoking instead of fully quit smoking.

**Table 1:** **Study outcomes, targets and measurements at T**_**0 **_**and T**_**1**_

**Primary outcomes**	**Target**	**Measurement**
BMI	≤25; or at least 5% reduction of bodyweight	Height: measured without shoes, by nurse Weight: measured without coat and shoes, by nurse
Waist circumference	♀ ≤ 88 cm, ♂ ≤ 102 cm	Measured by nurse with a measuring tape halfway between the lowest rib and the top of the hipbone around the abdomen, under (or without) clothing.
Physical activity	≥ 30 min. 5 times per week	Patient questionnaire: Physical Activity Scale for the Elderly (PASE; [20])
Systolic blood pressure	< 140 mmHg	Measured by nurse with an automatic sphygmomanometer. Patient is seated and both arms are measured. Measurement on the arm with the highest systolic blood pressure is repeated. The mean value of these both measurements is registered
Total cholesterol	≤ 5,0 mmol/l	Laboratory^*^
LDL cholesterol	≤ 2,5 mmol/l	Laboratory
HDL cholesterol	≥ 1,0 mmol/l	Laboratory
Diet	2 ounces of vegetables, 2 pieces of fruit, 20–35% energy intake of fat; < 10% energy intake of saturated fat	Patient questionnaire: Maastricht Voedingsvragenlijst[21]
**Secondary outcomes**		
Blood glucose	fasting glucose < 7 mmol/l	Laboratory
HbA1c (%)	< 53 mmol/mol (< 7%)	Laboratory
Smoking	fully quit	Patient questionnaire: Self report, one question
Self management		Patient questionnaire: Self report, five questions
Medication adherence	Full adherence	Patient questionnaire: Adapted Morisky Scale [22]
Quality of life		Patient questionnaire: MacNew Heart Disease Health-related Quality of Life Questionnaire [23]
Depression Anxiety		Patient questionnaire: Hospital Anxiety and Depression Scale (HADS; [24])

^*^ blood values are analysed by the laboratory in the medical centre the patient is treated, according to local standard practice.

In addition to the outcome measures, also gender, educational status, civil status, work status, the occurrence of life events in the past 12 months, ethnicity, cardiovascular history, treatment history, medication use and co morbidity are registered by the research assistant or nurse at baseline. Data are filled in using an electronic questionnaire.

Through a self developed (paper) patient questionnaire smoking, alcohol consumption, self care strategies in relation to coronary diseases and illness perception are measured. At six months, all measurements are repeated, except measurements of demographic characteristics. In addition, usual care received during the six months is registered through a patient dairy, and patients are asked about their experiences with the Hartcoach-programme.

### Randomisation and blinding

After informed consent has been obtained, each patient is randomly assigned to one of the two groups, regardless the medical centre the patient is treated. After the nurse has logged on to a specific portal, the patient is allocated computerized using a PHP RAND function to “Hartcoach + usual care” (experimental group) or to “usual care” alone (control group). The coordinating research centre informs the patient about the randomisation by a written letter. The measurements are performed by research-assistants or nurses in the treating hospital, who are blinded to allocation.

### Sample size calculation

Sample size calculation was based on one of the primary outcomes that were also used in the study of Vale et al (2003), namely total cholesterol. Based on the values found by Vale et al (2003) for total cholesterol (between groups difference of 0.36 mmol / L (95% confidence interval: 0.20-0.53 mmol / L) 160 patients per group would be needed to find statistical significant results (power of 0.80 and alpha of 0.05)[17]. In this study, a sample of 200 patients per group is chosen, to account for dropout.

### Statistical analysis

Data will be analyzed according to the intention-to-treat principle [25]. All patients, irrespective of completion of the Hartcoach-programme, will be included in the analysis. To determine the effect of the Hartcoach-programme, multilevel analysis will be used because the data have an intrinsic hierarchical nature. Patients are "nested" in hospitals and data are therefore not independent, violating a major assumption of traditional statistical procedures. The model we will use is a multilevel mixed model for repeated measures, a method in which all available data are adequately used: both the paired observations of patients who completed the intervention, as the details of the people who did not complete the Hartcoach-study [26].

Bivariate and multivariate analyses will be used to assess the effects of the intervention (compared to controls) on the individual risk factors cholesterol, body mass index, waist circumference, blood pressure, physical activity, diet, glucose, HbA1c and smoking, as well as on medication adherence, self-management, anxiety, depression and quality of life. The mean change in risk factors between 0 and 6 months is compared between intervention and control group. Age, gender, ethnicity, educational status, civil status, work status, the occurrence of life events in the past 12 months, cardiovascular history, medication use and co morbidity will be included as possible covariates. Data will be analyzed with the statistical packages Stata and MLWIN.

## Discussion

In this study, the effect of an evidence based telephone coaching programme (Hartcoach) in addition to usual care is evaluated on the reduction of individual risk factors of patients with CVD. Current programmes do not accomplish that patients with CVD change their lifestyle in a way that their individual risk factors for recurrent coronary events decrease. Hospital based rehabilitation programmes in the Netherlands differ strongly per hospital, both in content and extent. Furthermore, participation rates are rather low. The Hartcoach-programme is a pragmatic intervention that primes the patient to self management by adhering to medication and make relevant behaviour changes. Today’s policy is to enhance self management of patients as a means of improving long-term conditions and secondary prevention.

Patients who are not given the opportunity to follow a rehabilitation programme in the hospital or patients who are not able or motivated to adhere to a hospital based rehabilitation programme, may be reached by a home based programme. Another potential benefit of the Hartcoach-programme may be the positive influence the programme can have on medication adherence. In the Netherlands, statins are prescribed to almost every patient after an acute coronary event, but medication adherence to statins is known to be low. One-year persistence with statins has been estimated to be about 60% in patients with previous cardiovascular events [27-29].

A limitation of the study is the reliance on self report according to two of the primary outcomes, physical activity and diet. Since patients are not blinded to group allocation, this might have an impact on the results. However, both measurements, especially diet, are difficult and laborious to administer in a more reliable and valid manner. Another methodological weakness of the study is the fact that all patients receive usual care next to the Hartcoach-programme and some of the participant may follow a rather intensive rehabilitation programme. Additional effects of the Hartcoach-programme next to intensive rehabilitation may be too small to detect. However, in the Netherlands the Hartcoach-programme is implemented as an additional programme and was never intended to substitute usual care. Thereby, the amount of usual care and contingent cardiac rehabilitation is registered and can be taken into the analyses, while it is expected that there will also be a large number of patients who receive minimal care and support in managing their individual risk factors besides the Hartcoach-programme.

A large Dutch health insurance company (Achmea) already has introduced this programme to a limited number of hospitals and the Hartcoach-programme is offered to their clients. If positive results are found for the Hartcoach-programme, the implementation of the programme will be extended which will have implications for the management of many people with CVD. The Hartcoach-programme can be provided additional to existing services or as a "maintenance" programme for people after cardiac rehabilitation.

## Endnotes

^a^ During the study, we will try to find additional funding for a measurement at 12 months. This would not only provide a follow-up measurement but will also change the study design as the control group is invited to follow the Hartcoach-programme after completion of the six months measurement.

## Competing interests

The authors declare that they have no competing interests.

## Authors’ contributions

CV and LvD developed the study concept and aims. CL, HTJ, RJGP, LvD and CV co-wrote the study protocol. CL, LvD and CV are implementing the study protocol and overseeing the collection of data. CL drafted the study manuscript and all authors contributed to, read and approved the final manuscript.

## Pre-publication history

The pre-publication history for this paper can be accessed here:

http://www.biomedcentral.com/1471-2261/12/47/prepub
